# Online Health Information Seeking Among Patients With Chronic Conditions: Integrating the Health Belief Model and Social Support Theory

**DOI:** 10.2196/42447

**Published:** 2022-11-02

**Authors:** Yuxiang Chris Zhao, Mengyuan Zhao, Shijie Song

**Affiliations:** 1 School of Economics and Management Nanjing University of Science and Technology Nanjing China; 2 Fudan University Library Shanghai China; 3 Business School Hohai University Nanjing China; 4 School of Information Management Wuhan University Wuhan China

**Keywords:** health information seeking, patients with chronic conditions, health belief model, social support, critical health literacy

## Abstract

**Background:**

Chronic diseases are the leading causes of death and disability. With the growing patient population and climbing health care expenditures, researchers and policy makers are seeking new approaches to improve the accessibility of health information on chronic diseases while lowering costs. Online health information sources can play a substantial role in effective patient education and health communication. However, some contradictory evidence suggests that patients with chronic conditions may not necessarily seek online health information.

**Objective:**

This study aims to integrate 2 theories (ie, the health belief model and social support theory) and a critical health literacy perspective to understand online health information seeking (OHIS) among patients with chronic conditions.

**Methods:**

We used the survey method to collect data from online chronic disease communities and groups on social media platforms. Eligible participants were consumers with at least 1 chronic condition and those who have experience with OHIS. A total of 390 valid questionnaires were collected. The partial least squares approach to structural equation modeling was employed to analyze the data.

**Results:**

The results suggested that perceived risk (*t*=3.989, *P*<.001) and perceived benefits (*t*=3.632, *P*<.001) significantly affected patients’ OHIS. Perceived susceptibility (*t*=7.743, *P*<.001) and perceived severity (*t*=8.852, *P*<.001) were found to influence the perceived risk of chronic diseases significantly. Informational support (*t*=5.761, *P*<.001) and emotional support (*t*=5.748, *P*<.001) also impacted the perceived benefits of online sources for patients. In addition, moderation analysis showed that critical health literacy significantly moderated the link between perceived risk and OHIS (*t*=3.097, *P*=.002) but not the relationship between perceived benefits and OHIS (*t*=0.288, *P*=.774).

**Conclusions:**

This study shows that the health belief model, when combined with social support theory, can predict patients’ OHIS. The perceived susceptibility and severity can effectively explain perceived risk, further predicting patients’ OHIS. Informational support and emotional support can contribute to perceived benefits, thereby positively affecting patients’ OHIS. This study also demonstrated the important negative moderating effects of critical health literacy on the association between perceived risk and OHIS.

## Introduction

### Background

Chronic diseases are the leading global causes of death and disability. In the United States, 6 in 10 adults have 1 chronic disease, and 4 in 10 adults live with 2 or more chronic conditions [1]. According to the US Centers for Disease Control and Prevention (CDC), chronic diseases account for 3.8 trillion dollars in annual health care expenses in the United States [1]. In China, 3 chronic diseases (ie, cardiovascular diseases, cancer, and chronic respiratory diseases) were responsible for 80.7% of total deaths in 2019 [2]. Despite causing huge burdens, chronic diseases are influenced by several risk factors (eg, poor diet, physical inactivity, hyperlipidemia, and uncontrolled high blood pressure) that are generally preventable and manageable [3]. However, people living with chronic diseases often reported limited knowledge of the causes and consequences of their conditions [4]. Studies revealed that better informed patients are more likely to manage their chronic conditions, prevent exacerbations, and lower costs [5]. Due to the growing patient population and climbing health care expenditures, researchers and policy makers are seeking new approaches to improve the accessibility of health information on chronic diseases while lowering costs. Online health information sources can play a substantial role in facilitating effective patient education and health communication.

It is widely assumed that online health information seeking (OHIS) plays a significant role in the health management of patients with chronic diseases. Some evidence accords with this notion. For example, Madrigal and Escoffery [6] found that patients with chronic diseases are more likely to perform OHIS than those who are healthy and that patients with chronic diseases are more knowledgeable in OHIS. The phenomenon may be explained by the fact that health information needs trigger the OHIS process. Patients with chronic conditions have more explicit information needs than general consumers, including information on disease causes, lab testing results, and coping strategies [7-9]. Online sources are more convenient and accessible than formal health care services, so patients are assumed to perform OHIS frequently.

However, some contradictory evidence suggests that patients with chronic conditions may not necessarily seek health information. For example, McCloud and colleagues [10] conducted a mail-based survey in the United States and found that 1 in 3 cancer survivors intentionally avoided cancer-related information. Li et al [11] carried out a randomized field experiment in China and revealed that people avoid information on cancer and diabetes tests even when there is no monetary or transaction cost. A recent metareview concluded that health status is not a strong predictor of health information seeking [12]. Therefore, aside from health information needs, research questions of whether and why patients with chronic conditions seek health information online remain unresolved.

The existing research has applied many well-established theories to the portrayal of health behaviors among general consumers, such as the health belief model (HBM), social support theory, and health literacy. However, few attempts have been made to integrate these theories to understand health information behaviors comprehensively. Therefore, this paper aims to integrate 2 long-standing theories (ie, the health belief model and social support theory) and a critical health literacy perspective to understand online health information seeking among patients with chronic conditions.

### Research Model and Hypotheses

#### OHIS Among Patients With Chronic Conditions

Patients with chronic conditions have long-term health management demands; thus, many health experts call for patient activation, an ideal state wherein patients know how to manage their conditions, keep functioning, and prevent health declines [13]. The extrinsic needs related to health management (eg, to get better informed and to manage chronic conditions) and intrinsic motivations (eg, to seek social support) motivate patients to perform OHIS [14].

Moreover, the internet provides patients with a supportive environment for OHIS. Conventional online health information sources include general search engines [15], medical databases [16], online forums [17], and so forth. Recently, social media has become one of the most popular online health information sources among users [18]. Song et al [19,20] suggest that although many social media platforms were not intentionally designed for OHIS, the rich sets of technological affordances embedded in these platforms allow users to search for health-related content and facilitate user engagement. For example, YouTube empowers patients in chronic condition management [21], and TikTok has also been a critical channel for delivering chronic disease information [22].

#### HBM As an Explanatory Framework in Health Behavior Research

Historically, the HBM has been widely used to understand why patients engage in proactive health behaviors. Social psychologists developed the HBM in the 1950s to explain preventive health behaviors [23]. The model assumes that the intentions of taking proactive health actions rely more on individual beliefs about a particular condition than the objective facts of the condition [24]. According to the HBM, people’s proactive health behaviors are primarily determined by their *perceived susceptibility* to disease-related conditions, *perceived severity* of the consequences of disease-related conditions, *perceived benefits* of the behaviors in reducing the threats, and *perceived barriers* to the negative aspects of the health behaviors [25].

Numerous studies have investigated various health behaviors through the lens of HBM to contextualize health behaviors including a healthy diet [26], cancer screening [27], vaccination [28], medical help seeking [29], and preventive behaviors during epidemics [30]. For example, Hochbaum [31] applied the HBM when examining X-ray screening for tuberculosis and found that perceived susceptibility to tuberculosis and perceived benefits of screening varied across participants who had and had not received chest X-rays. More recently, Wong et al [28] employed the HBM to assess the acceptance of the COVID-19 vaccine and revealed that perceived severity of contracting COVID-19 and perceived benefits of receiving the vaccine positively predicted vaccine acceptance. Overall, these studies produced internally consistent results that provided fairly strong support for HBM and informed the subsequent use of HBM to understand health behaviors. Despite the intensive use of HBM in health and medical contexts, the model is less adopted to investigate health information behaviors. Given the considerable explanatory power of HBM in health sciences, this study will employ the HBM to investigate OHIS intentions among patients with chronic conditions.

Although the HBM does not specify the variable ordering, it implicitly purports the idea that perceived susceptibility and severity jointly lead to a perception of the risk of disease, and perceived benefits influence an individual’s assessment of the outcome of the proactive health behaviors [32]. As such, the risk-benefit consideration motivates the individual to take action. Noteworthily, the HBM does not provide rules of combinations of the constructs. For example, Harrison et al [33] did not include the cues to action and health motivation components in their analyses. Ahadzadeh et al [34] only included risk perceptions (ie, perceived susceptibility and perceived severity) when using the HBM. According to a recent systematic review [27], the risk-benefit aspect is the most frequently explored component in prior studies. Therefore, this study will also focus on the risk-benefit perspective.

The risk-benefit relationship posited by HBM has been partially examined in prior studies. For example, Ahadzadeh et al [34] found that risk perceptions had an indirect positive effect on Malaysian women’s online health-related internet use. Mou et al [35] observed that perceived benefits of online health websites, perceived susceptibility, and perceived severity of one’s health conditions were significant predictors of online health information seeking. Accordingly, our study proposes 2 hypotheses based on the parsimonious form of the HBM: (1) The OHIS of patients with chronic diseases is positively influenced by the perceived risk of chronic diseases (H1a) and the perceived benefits of performing OHIS (H1b); and (2) the perceived risk of chronic diseases of patients with chronic conditions is positively influenced by perceived susceptibility (H2a) and severity (H2b).

However, explicating the relationship between the HBM constructs cannot resolve all the theoretical limitations of the HMB. To overcome these constraints, researchers have often treated the HBM as an overarching framework [36] and combined its constructs with other theories [37]. For instance, Ahadzadeh et al [34] incorporated the HBM and the technology acceptance model to understand users’ online health-related internet behaviors. Mou et al [35] integrated the HBM, the extended valence framework, and the perspective of self-efficacy to explain users’ OHIS. Since prior work suggested that OHIS is associated with social support and health literacy [38], we will integrate the perspectives of social support and health literacy in this study.

#### Social Support in OHIS

Social support is often described as the comfort, help, or information that an individual obtains from others [39]. In offline settings, social support is often provided by friends and relatives [40]. In online environments, social media serves as an important source of social support for patients. For example, Zhang and He [41] found that people living with diabetes exchange medical and lifestyle information and provide and seek social support in Facebook groups. These Facebook diabetes groups share a broad variety of topics, such as nutrition, medications, blood glucose screening, and physical activity [42].

Social support has been extensively examined in health-related fields, with many studies finding positive associations between social support and people’s physical and mental health [43,44]. The benefits of social support are especially evident in patients’ self-management of chronic conditions [45]. However, despite its promising positive impacts, the mechanisms of how social support influences health behaviors remain underexplored. A couple of studies examined the direct associations between informational and emotional support and health behaviors or conditions. For example, Wang and Parameswaran [46] suggested that adequate online social support is correlated with better self-care behaviors of HIV patients. However, other studies revealed that the impacts of social support on health behaviors are mediated by different factors, such as health self-efficacy and health information seeking [47,48].

Although social support is a multifaceted concept with different subdimensions, informational and emotional supports are the most frequently studied aspects in the existing health literature [49]. Savolainen [50] found that dietary information seekers solicited emotional support in health blogs by describing their dieting problems, and readers responded by offering considerable informational and emotional support. Stellefson and Paige [42] surveyed the 34 largest diabetes support groups on Facebook and revealed that informational and emotional support exchanges were the 2 most common purposes for creating those groups. Therefore, this study will focus on these 2 main types of social support.

Regarding patients’ motivations for seeking online sources for social support, some researchers suggest a compensation view and posit that online sources can fulfill patients’ social support deficits from offline settings [51,52]. However, Guillory and Niederdeppe [53] found that patients who already had sufficient social support from families and friends were also likely to seek online health information. McKinley and Wright [47] assert that although their inconsistent findings cannot fully support the compensation view, they demonstrate that online social supports are helpful for the end users. Accordingly, we propose our third hypothesis (H3): The perceived benefits of online sources for patients with chronic conditions are positively influenced by online emotional support (H3a) and informational support (H3b).

#### Critical Health Literacy in OHIS

Health literacy refers to “the degree to which individuals can obtain, process, understand, and communicate about health-related information needed to make informed health decisions” [54]. According to Nutbeam [54], health literacy is a hierarchical concept consisting of multiple layers, depending on different levels of advancement of the literacy. While functional literacy refers to basic skills in reading and writing regarding health information, critical literacy refers to the advanced cognitive skills in analyzing health information critically.

Early studies treated health literacy as a holistic concept and found varied associations between health literacy and patients’ health behaviors [55]. However, many recent studies revealed that the different components of health literacy have different power in explaining health behaviors. For example, Heijmans and Waverijn [56] found that critical health literacy is related to self-management, but functional health literacy is not. Matsuoka and Tsuchihashi-Makaya [57] revealed similar findings that critical health literacy influences self-care and consulting behaviors but functional health literacy does not. Based on these findings, we argue that critical health literacy may influence patients’ information behaviors.

Moreover, prior studies suggested that patients with chronic conditions were concerned about the information quality, although they mostly agreed that online health information was easy to find [58]. These findings indicated that some patients might be knowledgeable about their health conditions [9] and thus are more critical when it comes to health information assessment. Therefore, we posit that the effects of the perceived risk and benefits of OHIS are moderated by critical health literacy. When patients have higher critical health literacy, they are more cautious when choosing online health information sources and may turn to authoritative sources such as offline health care providers. Thus, we propose the following hypotheses (H4): Critical health literacy negatively moderates the associations between perceived risk (H4a) and perceived benefits (H4b) and patients’ OHIS.

**Figure 1:**
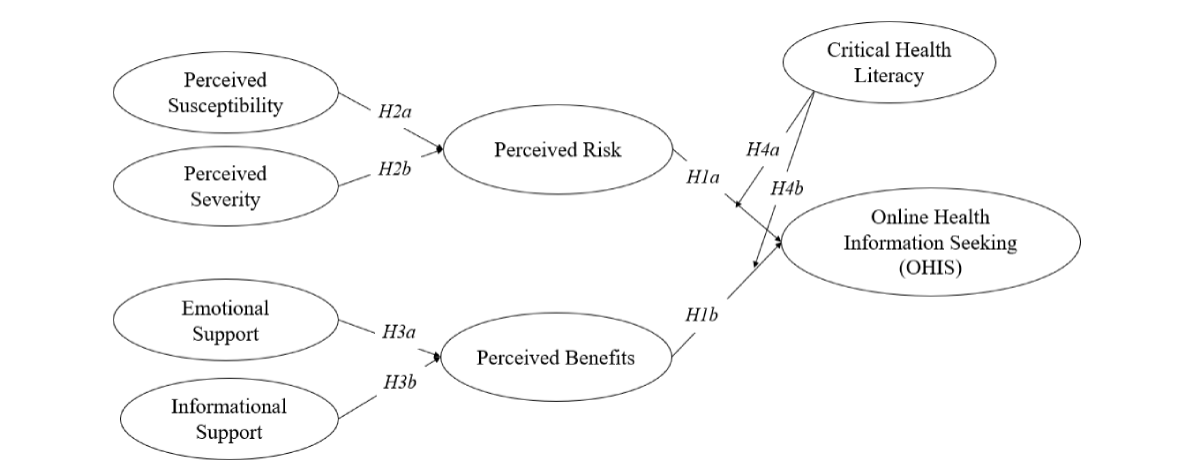
Proposed research model.

The research model and hypotheses are shown in Figure 1.

## Methods

### Measurement Instrument

Most of the construct items in this study were adapted from validated existing scales. Each item was measured following a 5-point Likert scale ranging from 1 (strongly disagree) to 5 (strongly agree). The 3 OHIS items were adapted from studies by Deng and Liu [48] and Li and Wang [59]. The 3 items measuring perceived risk were developed from Kahlor [60]. The perceived benefits scales were adjusted from McKinley and Wright [47]. The perceived susceptibility and severity were gauged based on studies by Ahadzadeh et al [34] and Shang and Zhou [61]. Measurements of emotional and informational support were derived from studies by Deng and Liu [48] and Li and Wang [59]. Three items for critical health literacy drew on the measurement developed by Ishikawa and Takeuchi [62] and converted into an index. The constructs and measures are shown in Table 1.

The questionnaire was formed in 2 stages. First, we used translation (from English to Chinese) and back-translation (from Chinese to English) techniques to design the questionnaire to ensure its reliability. Second, we invited 20 patients living with chronic disease to participate in a pilot survey. We gathered their feedback and suggestions during the completion of the initial questionnaire to further modify the questionnaire, which resulted in the final version of the questionnaire.

**Table 1:** Constructs and measures.

Constructs	Measures	References
Online health information seeking	OHIS^a^1: I want to seek health information often on the internet. OHIS2: I am willing to search the internet for relevant health information when I need it. OHIS3: I will seek health information on the internet before making health decisions.	Deng and Liu [48]; Li and Wang [59]
Perceived risk	PCR^b^1: I am constantly worried about my health condition. PCR2: I fear that my chronic condition would probably attack or worsen. PCR3: If my chronic disease attacks or worsens, it would have a serious impact on my work or life.	Kahlor [60]
Perceived benefits	PBF^c^1: Health information on the internet could be useful for me. PBF2: Health information on the internet could be helpful to me. PBF3: Health information on the internet could help me become familiar with health knowledge.	McKinley and Wright [47]
Perceived susceptibility	PSU^d^1: The health-related issues mentioned in the internet health information are likely to happen on me. PSU2: There is a good possibility that I will experience the health-related issues mentioned in the internet health information. PSU3: I am likely to contract the health-related issues mentioned in the internet health information.	Ahadzadeh et al [34]; Shang and Zhou [61]
Perceived severity	PSE^e^1: The consequences of the health-related issues mentioned in the internet health information may be serious for me. PSE2: Contracting the health-related issues mentioned in the internet health information would be likely to cause me major problems. PSE3: Suffering from the health-related issues mentioned in the internet health information is a serious problem for me.	Shang and Zhou [61]
Emotional support	ES^f^1: When faced with difficulties, some individuals on the internet comforted and encouraged me. ES2: When faced with difficulties, some individuals on the internet expressed interest in and concern for my well-being. ES3: When faced with difficulties, some individuals on the internet are on side with me.	Deng and Liu [48]; Li and Wang [59]
Informational support	IS^g^1: When faced with difficulties, some individuals on the internet would offer suggestions when I needed help. IS2: When faced with difficulties, some individuals on the internet would give me information to help me overcome the problem. IS3: When faced with difficulties, some individuals on the internet would help me discover the cause and provide me with suggestions.	Li and Wang [59]
Critical health literacy	CHL^h^1: Since being diagnosed with chronic diseases, I have considered whether the information was applicable to my situation. CHL2: Since being diagnosed with chronic diseases, I have considered the credibility of the information. CHL3: Since being diagnosed with chronic disease, I have checked whether the information was valid and reliable.	Ishikawa and Takeuchi [62]

^a^OHIS: online health information seeking.

^b^PCR: perceived risk.

^c^PBF: perceived benefit.

^d^PSU: perceived susceptibility.

^e^PSE: perceived severity.

^f^ES: emotional support.

^g^IS: informational support.

^h^CHL: critical health literacy.

### Ethics Approval

This study was approved by the Institutional Review Boards of the School of Economics and Management of the Nanjing University of Science and Technology (20201101).

### Data Collection

The questionnaire was distributed from 2 main channels. First, we recruited participants through online chronic disease health communities. Five typical online health forums (ie, diabetes, hypertension, chronic gastritis, hyperlipoidemia, and rhinitis) were chosen in each of the leading Chinese communities (ie, Baidu Tieba and Douban groups). We also distributed the questionnaire through chronic disease health groups on general social media platforms (eg, WeChat). Eligible participants were consumers with at least 1 chronic condition who sought health information online during the past 12 months. The questionnaire contained a consent form that included the details of the study. Participants who agreed to the consent continued to the questionnaire. Each participant received a cash incentive of 5 renminbi (RMB) (about US $0.8) after completing the questionnaire. We received 426 questionnaires from October 18 to 29, 2021. After eliminating incomplete and invalid questionnaires by applying the eligibility criteria, we finally obtained a sample consisting of 390 valid responses.

### Statistical Analysis

The respondents’ characteristics are illustrated in Table 2. Of the participants, 64.1% (n=250) were male, and 35.9% (n=140) were female. The age coverage was relatively broad, comprising young people under the age of 20 and older adults above the age of 60 years. Respondents’ places of residence were relatively balanced, with 46.7% (n=182) of participants living in urban areas and 53.3% (n=208) living in rural areas. Approximately half (n=192, 49.2%) of the participants had college degrees. In terms of health status, 38.5% (n=150) of the participants reported feeling normal, 25.6% (n=100) felt bad, and 35.9% (n=140) felt good or very good. Participants reported various types of chronic conditions. Chronic gastritis (n=146, 37.4%) was the most frequently mentioned condition, followed by diabetes (n=114, 29.2%) and hyperlipidemia (n=98, 25%). About half (n=193, 49.5%) of the participants had 1 chronic condition, 31.79% (n=124) had 2, and 4% (n=17) had 4 or more conditions.

We also measured the types of health information that participants sought using a typology from Zhao and Zhao [38]. Participants most frequently sought health information about disease symptoms (n=209, 53.6%), medical resources (n=201, 51.5%), and health prevention (n=199, 51%). Additionally, we counted the online health information sources that the participants used. Medical and health apps (n=187, 48%) were the most frequently reported online health information source, followed by social question-and-answer platforms (n=179, 46%) and short video platforms (n=174, 44.6%). Regarding OHIS frequency, all the participants reported they had sought online health information at least once during the past 6 months, and 39.5% (n=154) participants reported that they had sought online health information relatively often or very frequently.

**Table 2:** Characteristics of respondents.

Measure and item			Value, n (%)		
**Sex**					
	Male	250 (64.1)			
	Female	140 (35.9)			
**Age**					
	＜20	13 (3.33)			
	20-29	131 (33.6)			
	30-39	137 (35.1)			
	40-49	58 (14.9)			
	50＜59	35 (9)			
	≥60	16 (4.1)			
**Place of residence**					
	Urban	182 (46.7)			
	Rural	208 (53.3)			
**Education level**					
	Junior high school or below	58 (14.9)			
	Senior high school	98 (25.1)			
	Technical secondary school	42 (10.8)			
	Associate degree	72 (18.5)			
	Bachelor’s degree	103 (26.4)			
	Master’s degree	17 (4.4)			
**Monthly income (RMB^a^)**					
	＜1500	17 (4.4)			
	1500-2999	55 (14.1)			
	3000-3999	112 (28.7)			
	4000-4999	68 (17.4)			
	5000-5999	71 (18.2)			
	6000-6999	29 (7.4)			
	≥7000	38 (9.7)			
**Profession**					
	Currently in health care profession	46 (11.8)			
	Past worked in health care profession	208 (53.3)			
	Never worked in health care profession	136 (34.9)			
**Health status**					
	Very bad	17 (4.4)			
	Relatively bad	83 (21.3)			
	Normal	150 (38.5)			
	Relatively good	100 (25.6)			
	Very good	40 (10.3)			
**Type of chronic disease**					
	Chronic gastritis	146 (37.4)			
	Diabetes	114 (29.2)			
	Hyperlipoidemia	98 (25.1)			
	Hypertension	76 (19.5)			
	Rhinitis	72 (18.5)			
	Rheumatism	62 (15.9)			
	Lumbar disc bulging	37 (9.5)			
	Asthma	33 (8.5)			
	Chronic conjunctivitis	33 (8.5)			
	Other	10 (2.6)			
**Number of chronic diseases**					
	1	193 (49.5)			
	2	124 (31.8)			
	3	56 (14.4)			
	4	11 (2.8)			
	＞4	6 (1.5)			
**Type of health information**					
	Disease symptoms	209 (53.6)			
	Medical resource	201 (51.5)			
	Health prevention	199 (51)			
	Medication/treatment	111 (28.5)			
	Health promotion	94 (24.1)			
	Other	4 (1)			
**Source of health information**					
	Medical and health apps				187 (48)
	Social question-and-answer platforms				179 (45.9)
	Short video platforms				174 (44.6)
	Social platforms				122 (31.3)
	Search engines				111 (28.5)
	News clients				56 (14.4)
	Other				8 (2.1)
**Frequency of searching**					
	Occasionally			83 (21.3)	
	Sometimes			153 (39.2)	
	Relatively often			127 (32.6)	
	Very frequently			27 (6.9)	

^a^RMB: renminbi.

## Results

### Approach

We employed a partial least squares (PLS) approach to structural equation modeling (SEM) on testing the proposed model. Previous studies have shown that the PLS-SEM method is suitable for testing theoretically constructed models [63] and validating relatively complex models [64]. In addition, PLS-SEM can deal with nonnormally distributed samples, which is advantageous when processing relatively small sample sizes [65]. We used SmartPLS 3 software (SmartPLS GmbH) to analyze the data and test the structural model.

### Measurement Model

Drawing on Shang and Zhou [61], we adopted reliability, convergent, and discriminant validity to evaluate the measurement model. Table 3 reports the reliability and convergence validity results. The reliability was judged based on the Cronbach alpha and composite reliability values. The results show that all Cronbach alpha and composite reliability values were greater than the proposed threshold of 0.7 [66], indicating qualified reliability. The convergence validity was examined by the values of average variance extracted (AVE). The results show that AVEs were higher than the recommended value of 0.5 [67], and all indicator loadings exceeded the threshold of 0.7, suggesting satisfactory convergence validity.

The discriminant validity was checked by testing both the Fornell-Larcker criteria [68] and the heterotrait-monotrait ratio (HTMT) [69]. Table 4 suggested that the square root of AVE values for each construct exceeded all its correlation coefficients with other constructs, indicating promising discriminant validity [68]. Moreover, all HTMT values were below the recommended value of 0.85 (Table 5), suggesting good discriminant validity [69]. The foregoing results verify the discriminant validity of all the constructs in our study.

**Table 3:** Reliability and convergence validity.

Constructs and items			Indicator loading		Cronbach alpha		Composite reliability		AVE^a^
**Perceived susceptibility**					.814		.890		.729
	PSU^b^1	.881							
	PSU2	.795							
	PSU3	.883							
**Perceived severity**					.852		.910		.772
	PSE^c^1	.888							
	PSE2	.861							
	PSE3	.886							
**Informational support**					.831		.898		.747
	IS^d^1	.883							
	IS2	.832							
	IS3	.878							
**Emotional support**					.856		.913		.777
	ES^e^1	.896							
	ES2	.861							
	ES3	.888							
**Perceived risk**					.835		.901		.752
	PCR^f^1	.882							
	PCR2	.834							
	PCR3	.885							
**Perceived benefits**					.821		.894		.737
	PBF^g^1	.867							
	PBF2	.834							
	PBF3	.874							
**Online health information seeking**					.824		.895		.740
	OHIS^h^1	.881							
	OHIS2	.823							
	OHIS3	.874							

^a^AVE: average variance extracted.

^b^PSU: perceived susceptibility.

^c^PSE: perceived severity.

^d^IS: informational support.

^e^ES: emotional support.

^f^PCR: perceived risk.

^g^PBF: perceived benefit.

^h^OHIS: online health information seeking.

**Table 4:** Discriminant validity (Fornell-Larcker criterion)^a^.

Constructs	1	2	3	4	5	6	7
1. Emotional support	.881	—	—	—	—	—	—
2. Online health information seeking	.571	.860	—	—	—	—	—
3. Informational support	.621	.526	.864	—	—	—	—
4. Perceived benefits	.684	.660	.676	.858	—	—	—
5. Perceived risk	.526	.578	.461	.585	.867	—	—
6. Perceived severity	.522	.576	.460	.582	.717	.879	—
7. Perceived susceptibility	.513	.529	.442	.488	.698	.629	.854

^a^Values on the diagonal represent the square root of average variance extracted (AVE) for each construct.

**Table 5:** Discriminant validity (heterotrait-monotrait ratio).

Items	1	2	3	4	5	6	7
1. Emotional support							
2.Online health information seeking	.677						
3. Informational support	.735	.633					
4. Perceived benefits	.816	.799	.815				
5. Perceived risk	.624	.692	.550	.707			
6. Perceived severity	.610	.687	.544	.694	.848		
7. Perceived susceptibility	.615	.643	.537	.598	.844	.754	

### Structural Model

**Figure 2:**
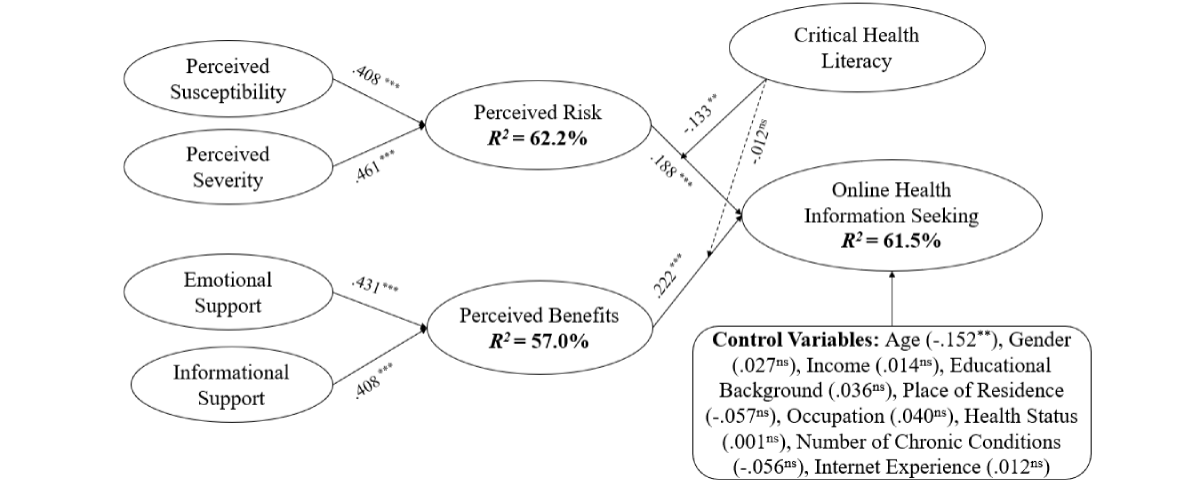
Structural model results. ns: nonsignificant. ***P<.001, **P<.01, and *P<.05.

We adopted standard bootstrap in SmartPLS 3 on 5000 bootstrapping samples to examine the structural model’s path coefficients and corresponding significance levels. Figure 2 shows the results of the PLS-SEM analysis, where perceived risk, perceived benefits, and online health seeking behavior are explained by the independent variables with variance values of 62.2%, 57%, and 61.5%, respectively, indicating a good explanation of the structural model.

The hypotheses testing results (Table 6) show that perceived risk (*β*=.188, *P*<.001) and perceived benefits (*β*=.222, *P*<.001) have significant positive effects on OHIS, supporting both H1a and H1b. As for health beliefs, perceived susceptibility (*β*=.408, *P*<.001) and perceived severity (*β*=.461, *P*<.001) significantly influence perceived risk, indicating that both H2a and H2b are supported. Concerning social support, both emotional support (*β*=.431, *P*<.001) and informational support (*β*=.408, *P*<.001) have positive effects on perceived risk, supporting H3a and H3b. Moreover, we tested the moderating effects of critical health literacy. The results show that critical health literacy (*β*=−.133, *P*=.002) has negative moderating effects on the relationship between perceived risk and OHIS, which supports H4a. However, critical health literacy cannot significantly moderate the relationship between perceived benefits and OHIS (*β*=−.012, *P*=.774). Therefore, H4b is not supported.

**Table 6:** Hypotheses testing results.

Hypotheses	Paths	Path coefficients	*t*-statistic	*P* value	Hypothesis validation
H1a	PCR -> OHIS	.188	3.989	<.001	Supported
H1b	PBF -> OHIS	.222	3.632	<.001	Supported
H2a	PSU -> PCR	.408	7.743	<.001	Supported
H2b	PSE -> PCR	.461	8.852	<.001	Supported
H3a	ES -> PBF	.431	5.748	<.001	Supported
H3b	IS -> PBF	.408	5.761	<.001	Supported
H4a	PCR×CHL -> OHIS	−.133	3.097	.002	Supported
H4b	PBF×CHL -> OHIS	−.012	0.288	.774	Not supported

## Discussion

### Principle Findings

In this study, we investigated the effects of perceived risk and perceived benefits on OHIS among patients with chronic conditions. Based on HBM, we examined the influencing factors of perceived risk using 2 antecedents: perceived susceptibility and perceived severity. Additionally, drawing on social support theory, we explored the impact of informational and emotional support on perceived benefits of patients’ OHIS. This study also focused on critical health literacy and how it moderates the effects of perceived risk and perceived benefits on OHIS. We proposed a research model by integrating the aforementioned theories and developed corresponding measurement instruments. Data were collected from online chronic disease communities and social media groups using the survey method and analyzed using the PLS-SEM method.

The results suggested that perceived risk (*t*=3.989, *P*<.001) and perceived benefits (*t*=3.632, *P*<.001) significantly affected patients’ OHIS. Perceived susceptibility (*t*=7.743, *P*<.001) and perceived severity (*t*=8.852, *P*<.001) were found to significantly influence the perceived risk of chronic diseases. Informational support (*t*=5.761, *P*<.001) and emotional support (*t*=5.748, *P*<.001) also impacted the perceived benefits of online sources for patients. In addition, moderation analysis showed that critical health literacy significantly moderates the relationship between perceived risk and OHIS (*t*=3.097, *P*=.002) but not the relationship between perceived benefits and OHIS (*t*=0.288, *P*=.774).

### Implications

This study makes contributions to both theory and practice. From a theoretical perspective, we extend the HBM into information behavior research by integrating it with the social support theory. The HBM suggests that belief in health risk predicts the likelihood of engaging in health-related behaviors [37]. Prior work shows that individuals with higher perceived risk have a stronger motivation to perform health-related behaviors and change their health conditions [34,70]. Among them, patient-initiated OHIS can undoubtedly meet patients’ health information needs and promote positive health information behaviors to a certain extent. In addition to patients’ spontaneous health beliefs, this paper argues that social determinants of health can largely contribute to patients’ health information behaviors—social support as an intermediary social determinant predicts patients’ OHIS. We believe this assertion can simultaneously enrich the HBM and literature on health information behaviors. Our empirical study confirms the validity of this extension. Wilson [71] suggested that the disciplines of health and medical sciences and information sciences share a prominent common interest in information behavior research, and the flows of ideas and theories from the community of interest would also benefit information behavior research.

Additionally, we contextualize health literacy in chronic diseases by proposing and testing how critical health literacy moderates the relationship between health beliefs and social support to patients’ OHIS. Prior work has explored the measurement of critical health literacy for patients with chronic diseases and the impact on self-management of health [56,72]. However, few studies have analyzed the impact of critical health literacy on OHIS. Our analysis contributes to the literature by uncovering a negative moderating effect between perceived risk and OHIS. We speculated that patients with higher critical health literacy may also be more capable in health information seeking and source selections. When patients with higher critical health literacy perceive a greater health risk, they may not necessarily search for health information on the internet and social media, given the general information quality concerns with online sources; instead, they are likely to seek more professional medical advice and visit doctors directly. This finding allows us to reexamine the compound influences of OHIS and seek more theoretical support from a psychological perspective.

From a practical perspective, this study suggests that online health communities should provide sufficient social support to patients and create a reciprocal virtual community. This social support can come from high-quality content created by professionals or emotional support generated by the mutual help between patient-patient and doctor-patient interactions. Meanwhile, online health communities should encourage surrogate health information seeking among patients and enhance the sense of belonging to the virtual community through gamification incentives and participatory design methods.

Finally, online health platforms need to better segment their users by providing targeted professional services to differentiated patients according to their varied health literacy levels instead of the traditional demographic profiles. Patients can become well informed about their health conditions and evolve into “expert patients.” Expert patients with high health literacy usually have higher health information quality standards and prefer to go to offline professional medical institutions for consultation. Therefore, online health communities could consider inviting health care experts to carry out freemium consultations with more specialized, personalized, and accurate services to retain patients with higher critical health literacy and enhance their stickiness and loyalty to online health platforms.

### Limitations and Future Work

This study has several limitations. First, the underlying influence mechanism between the 2 theories (ie, the health belief model and the social support theory) needs to be further empirically demonstrated. Future research could consider health beliefs as mediating constructs to unravel the effects of social determinants of health on individuals’ perceived risks and benefits and further draw on social cognitive theory to empirically explore this mediating effect.

Second, we identified the moderating effect of critical health literacy in OHIS; however, the moderation analysis indicates that more contextualized measures are needed to validate the working mechanisms of critical health literacy. Future research needs to uncover how critical health literacy moderates the patients’ OHIS intentions. Additionally, future research could further empirically analyze the constituent domains of critical health literacy [72] in terms of the dimensions of the constructs and how they are measured. Furthermore, researchers may also consider a randomized controlled trial to explore the effects of improved critical health literacy on OHIS.

Third, the generalizability may be limited as our sample is restricted to chronic disease patients in China. Our findings may not be applicable to other countries, regions, and contexts. Future work may conduct cross-cultural and cross-national comparisons to better generalize this study’s results. Moreover, this is a cross-sectional study; due to the diversity of chronic diseases and the dynamic nature of chronic conditions, more longitudinal studies are needed in the future to reveal the dynamic effects of changes in health beliefs and social support on OHIS among patients with chronic diseases. Experience sampling methods and action research approaches are recommended to improve the validity of the research through multiwave data collection.

### Conclusions

This paper contributes to the literature on OHIS by integrating the HBM and the social support theory. The integrated model suggested that health beliefs and social support positively impact OHIS among patients with chronic diseases. In particular, perceived susceptibility and severity can positively impact perceived risk, further influencing patients’ OHIS. Informational support and emotional support can contribute to perceived benefits, further positively affecting patients’ OHIS. This study also demonstrated critical health literacy’s important negative moderating effects on the association between perceived risk and OHIS. Theoretical and practical implications for leveraging OHIS for patients with chronic diseases were also provided.

## Abbreviations

CDC: Centers for Disease Control and Prevention
HBM: health belief model
HTMT: heterotrait-monotrait ratio
OHIS: online health information seeking
PLS: partial least square
RMB: renminbi
SEM: structural equation modeling

## Data Availability

The data sets generated and analyzed during this study are available from the corresponding author on reasonable request.
